# Carbonic Anhydrase Generates CO_2_ and H^+^ That Drive Spider Silk Formation Via Opposite Effects on the Terminal Domains

**DOI:** 10.1371/journal.pbio.1001921

**Published:** 2014-08-05

**Authors:** Marlene Andersson, Gefei Chen, Martins Otikovs, Michael Landreh, Kerstin Nordling, Nina Kronqvist, Per Westermark, Hans Jörnvall, Stefan Knight, Yvonne Ridderstråle, Lena Holm, Qing Meng, Kristaps Jaudzems, Mitchell Chesler, Jan Johansson, Anna Rising

**Affiliations:** 1 Department of Anatomy, Physiology and Biochemistry, Swedish University of Agricultural Sciences, Uppsala, Sweden; 2 Institute of Biological Sciences and Biotechnology, Donghua University, Shanghai, People's Republic of China; 3 Department of Physical Organic Chemistry, Latvian Institute of Organic Synthesis, Riga, Latvia; 4 Department of Medical Biochemistry and Biophysics, Karolinska Institutet, Stockholm, Sweden; 5 Department of Neurobiology, Care Sciences and Society (NVS), Karolinska Institutet, Stockholm, Sweden; 6 Department of Immunology, Genetics and Pathology, Uppsala University, Uppsala, Sweden; 7 Department of Cell and Molecular Biology, Uppsala University, Uppsala, Sweden; 8 Departments of Neurosurgery, Physiology and Neuroscience, New York University School of Medicine, New York, New York, United States of America; 9 Institute of Mathematics and Natural Sciences, Tallinn University, Tallinn, Estonia; Brandeis University, United States of America

## Abstract

Spider silk fibers are produced from soluble proteins (spidroins) under ambient conditions in a complex but poorly understood process. Spidroins are highly repetitive in sequence but capped by nonrepetitive N- and C-terminal domains (NT and CT) that are suggested to regulate fiber conversion in similar manners. By using ion selective microelectrodes we found that the pH gradient in the silk gland is much broader than previously known. Surprisingly, the terminal domains respond in opposite ways when pH is decreased from 7 to 5: Urea denaturation and temperature stability assays show that NT dimers get significantly stabilized and then lock the spidroins into multimers, whereas CT on the other hand is destabilized and unfolds into ThT-positive β-sheet amyloid fibrils, which can trigger fiber formation. There is a high carbon dioxide pressure (pCO_2_) in distal parts of the gland, and a CO_2_ analogue interacts with buried regions in CT as determined by nuclear magnetic resonance (NMR) spectroscopy. Activity staining of histological sections and inhibition experiments reveal that the pH gradient is created by carbonic anhydrase. Carbonic anhydrase activity emerges in the same region of the gland as the opposite effects on NT and CT stability occur. These synchronous events suggest a novel CO_2_ and proton-dependent lock and trigger mechanism of spider silk formation.

## Abstract 2

Mapping the conditions of spider silk proteins along the silk gland, and combining with molecular studies, reveals a pH controlled switch between lock and trigger forms, providing insights into spider silk formation.

## Author Summary

The spinning process of spider silk is crucial for making webs or other complex constructions to catch spider's prey. The main components of the silk are spidroins, which are large and repetitive proteins that have conserved nonrepetitive terminal domains (NT and CT). Spiders manage both to store the highly aggregation-prone spidroins in solution at extreme concentrations in the silk glands and then to rapidly convert these spidroins into a solid fiber within fractions of a second as they spin fibres. This process has been extensively studied and is thought to involve a pH gradient, but how this pH gradient is generated and maintained was not resolved. Here, we measured the pH at locations along the ampullate gland and determined that the pH decreases to 5.7 in the middle of the spinning duct. We also observed that the carbon dioxide pressure is simultaneously increased and that its accumulation may affect the stability of CT. We find that active carbonic anhydrase (CA) is crucial to maintain the pH gradient along the gland. Detailed molecular studies of NT and CT under the disparate conditions present along the gland revealed a lock and trigger mechanism whereby in more neutral pH conditions, precocious spidroin aggregation is prevented, and when in more acidic pH conditions, NT dimers firmly interconnect the spidroins and the CT unfolds into β-sheet nuclei that can trigger rapid polymerization of the spidroins. We conclude that this mechanism enables temporal and spatial control of silk formation and may be harnessed in attempts to produce artificial silk replicas.

## Introduction

Spider silk fibers contain regions of crystalline and noncrystalline β-sheets, which mediate mechanical stability [1]. In contrast, the soluble spidroins (dope) stored in the tail and sac of major and minor ampullate silk glands [2] exhibit unordered and helical conformations [3]. How spiders rapidly convert the dope into a solid fiber at a defined site of the S-shaped duct has been extensively studied [4]–[8], but major questions are unresolved: First, how is the pH gradient in the gland generated and maintained? Second, what is the pH at the phase transition in the duct? The pH in the major ampullate gland has been shown to decrease from 7.2 in the proximal parts of the sac to 6.3 in the beginning of the duct [7], but it has also been proposed that the gradient goes from 6.9 in the sac to 6.3 in the third limb of the duct [6]. Third, how are the terminal domains affected by the conditions in the duct at a molecular level, and in particular, do they, as proposed [4],[5], act in similar manners? Documented pH-dependent effects at a molecular level include that the N-terminal domain (NT) dimerizes at pH 6 [9]–[11], but pH-induced structural changes of the C-terminal domain (CT) have only been observed at pH 2 [4]. Here we address these questions and unravel novel physiological mechanisms for regulated spider silk formation.

## Results and Discussion

**Figure 1:**
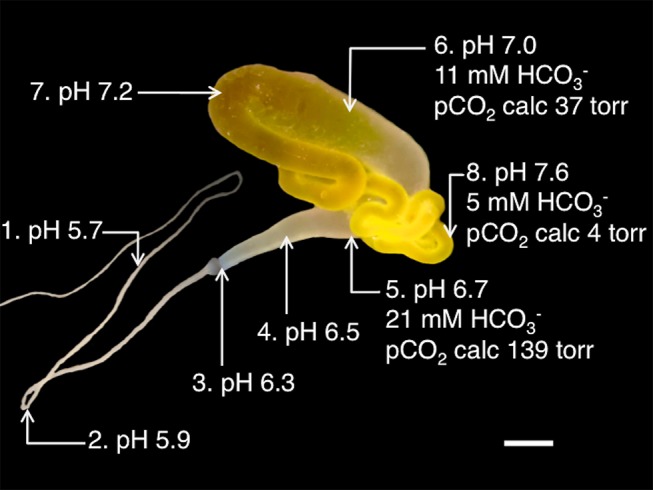
pH, bicarbonate, and carbon dioxide in major ampullate glands. Photograph of a major ampullate gland in which measured pH and HCO_3_^−^ values and calculated pCO2 values at different locations are indicated. See Table 1 for details of ISM measurements. Scale bar, 1 mm.

By use of concentric ion selective microelectrodes (ISMs) [12] we determined the pH in the major ampullate gland of *Nephila clavipes*, from the proximal part of the tail to the middle part of the second limb of the duct. Concentrations of CO_3_^2−^ were also determined at locations where pH was high enough to allow reliable measurements, and used to calculate HCO_3_^−^ concentrations. We found that the pH decreases from 7.6±0.1 (*n* = 11) in the proximal tail to 5.7±0.0 (*n* = 6) in the second limb of the duct and that HCO_3_^−^ concentration increases from 5 mM in the proximal tail to 21 mM in the distal part of the sac (Figure 1 and Table 1). With these values in the Henderson–Hasselbalch equation, the carbon dioxide pressure (pCO_2_) could be calculated and was found to increase along the gland (Figure 1). We observed that the intraluminal pH at different locations did not change despite superfusion of the gland with an elevated pCO_2_. This indicates that the epithelium of the major ampullate gland does not allow permeation of CO_2_, a phenomenon previously described for parietal and chief cells in gastric glands [13]. The concentrations of K^+^, Na^+^, and Cl^−^ in the sac were determined to be 6, 192, and 164 mM, respectively, using concentric ISMs (Table 1).

**Table 1:** pH and ion concentrations in major ampullate glands.

Location	pH ± SD	HCO_3_^−^ (mM) ± SD	K^+^ (mM) ± SD	Na^+^ (mM) ± SD	Cl^−^ (mM) ± SD
1	5.7±0.0 (*n* = 6)	—	—	—	—
2	5.9±0.1 (*n* = 6)	—	—	—	—
3	6.3±0.0 (*n* = 4)	—	—	—	—
4	6.5±0.1 (*n* = 5)	—	—	—	—
5	6.7±0.1 (*n* = 19)	21±4 (*n* = 13)	—	—	—
6	7.0±0.1 (*n* = 27)	11±4 (*n* = 14)	6±1 mM (*n* = 8)	192±8 mM (*n* = 8)	164±7 mM (*n* = 6)
7	7.2±0.1 (*n* = 8)	—	—	—	—
8	7.6±0.1 (*n* = 11)	5±3 (*n* = 5)	—	—	—

Summary of mean ± SD for pH values, HCO_3_^−^, K^+^, Na^+^, and Cl^−^ concentrations measured at different locations in *N. clavipes* major ampullate glands. SD, standard deviation. *n* =  number of glands used. — indicates no measurements at this location. Locations are indicated in Figure 1.

**Figure 2:**
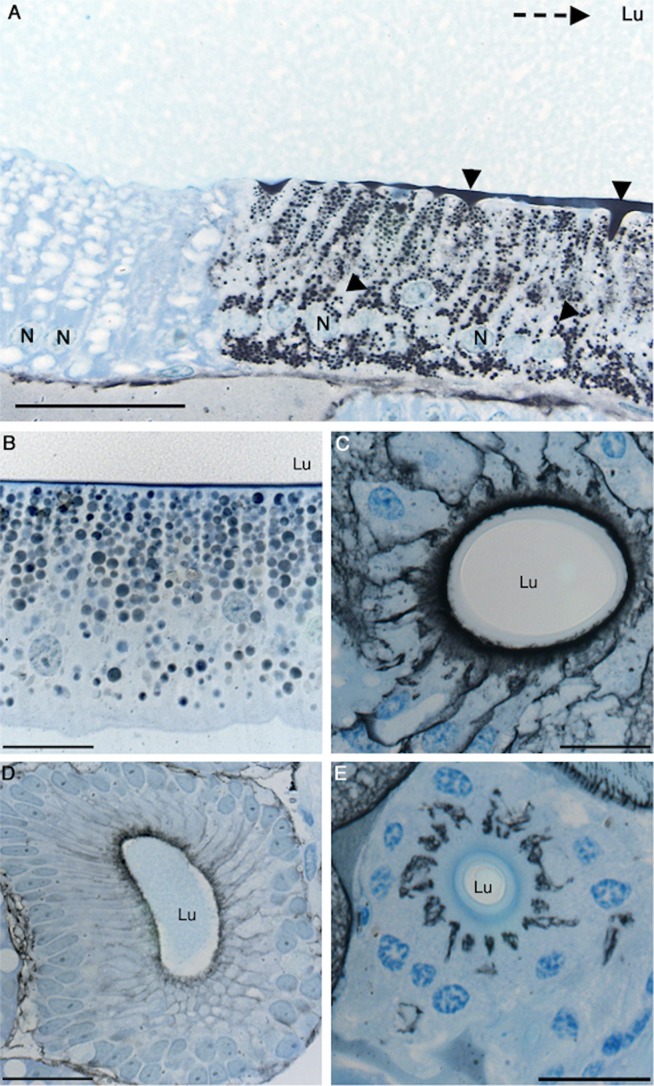
CA in spider silk glands. CA activity and Azure blue staining of histological sections from (A) the sac of a *Tegenaria sp*. major ampullate gland, (B) *E. australis* minor ampullate gland, (C) *A. diadematus* aggregate gland duct, (D) *Tegenaria sp*. tubuliform gland, and (E) the third limb of the duct of an *A. diadematus* major ampullate gland. Black precipitates represent CA activity (arrow heads). In (A) the glandular lumen is labeled and the dotted arrow points towards the duct. Nuclei are indicated by (N) and the lumen by (Lu). Scale bar, (A) 50 µm and (B–E) 20 µm.

The observation of simultaneously decreasing pH and increasing HCO_3_^−^ and CO_2_ concentrations from the proximal to the distal parts of the gland (Figure 1) suggested that carbonic anhydrase (CA) could be involved through catalysing the conversion of H_2_O + CO_2_ ↔H^+^+ HCO_3_^−^. By use of a histochemical method [14] we could indeed identify abundant CA activity in intracellular vesicles and at the apical cell membrane of the epithelium in the distal part of the major and minor ampullate sacs and ducts, as well as in aggregate gland ducts and tubuliform glands (Figure 2A–E). The site in the major ampullate epithelium where CA was found to emerge (Figure 2A) exactly coincides with the location where the glandular epithelium ceases to produce spidroins [15]. To investigate whether CA is responsible for generating and maintaining the pH gradient, we immersed freshly dissected *N. clavipes* major ampullate glands in buffers containing methazolamide, a membrane-permeable CA inhibitor [16]. Exposure to methazolamide collapsed the pH gradient, and pH levelled out to approximately 7 in the tail and sac. The gradient could subsequently be restored by removing the methazolamide (Table 2). Thus, the pH gradient in the major ampullate gland is dependent on active CA. Because CA activity was found in the epithelium of the distal major ampullate duct (Figure 2E), where also proton pumps are present [17], the pH may well continue to drop along the entire duct—that is, below pH 5.7 now measured half-way through the duct. This needs to be experimentally verified, as the extremely small inner diameter in the second half of the duct (<20 µm) did not allow measurements with the currently used ISMs.

**Table 2:** Effect of methazolamide (MTZ) on the pH gradient.

Location	pH – MTZ	pH + MTZ	pH After Washing
5	6.7	7.0	6.8
6	7.0	7.1	7.0
8	7.6	7.2	7.5

pH values in *N. clavipes* major ampullate glands before MTZ treatment (pH – MTZ), during MTZ treatment (pH + MTZ, in the presence of 0.1 mM MTZ for 1 h) and after washing for 30 min (pH after washing). Locations are indicated in Figure 1.

**Figure 3:**
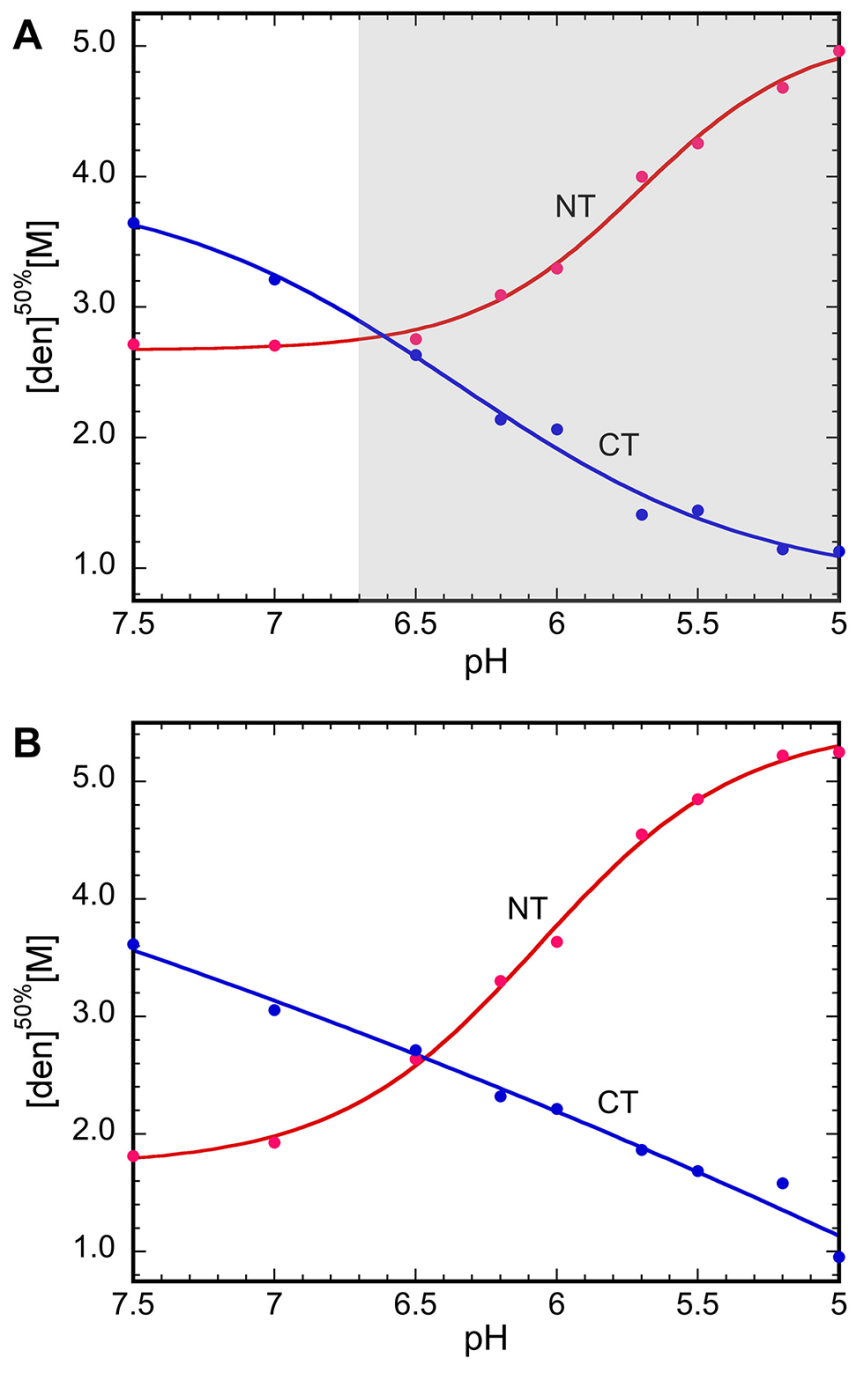
NT and CT respond differently to lowered pH. Stability of NT and CT (from *A. ventricosus*) in (A) 20 mM HEPES/MES buffer with 154 mM NaCl and (B) the same buffer without NaCl, measured with Trp fluorescence and CD spectroscopy at 222 nm, respectively, presented as urea concentrations for apparent half-denaturation ([den]^50%^, see Materials and Methods for details on how [den]^50%^ was determined) as a function of pH. The pH region in which CA activity is found in major ampullate glands is indicated by a shaded area in (A).

**Figure 4:**
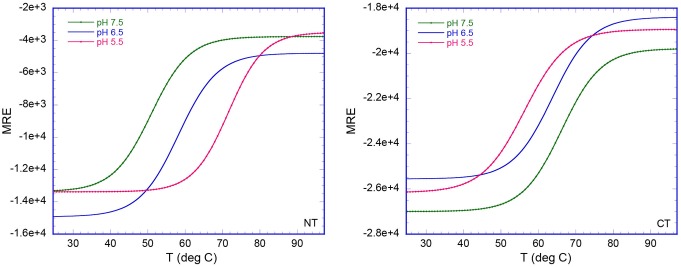
Temperature-induced unfolding of NT and CT. The CD signal was measured at 222 nm at pH 7.5, 6.5, and 5.5 and converted to mean residue ellipticity (MRE) in deg × cm^2^/dmol.

**Figure 5:**
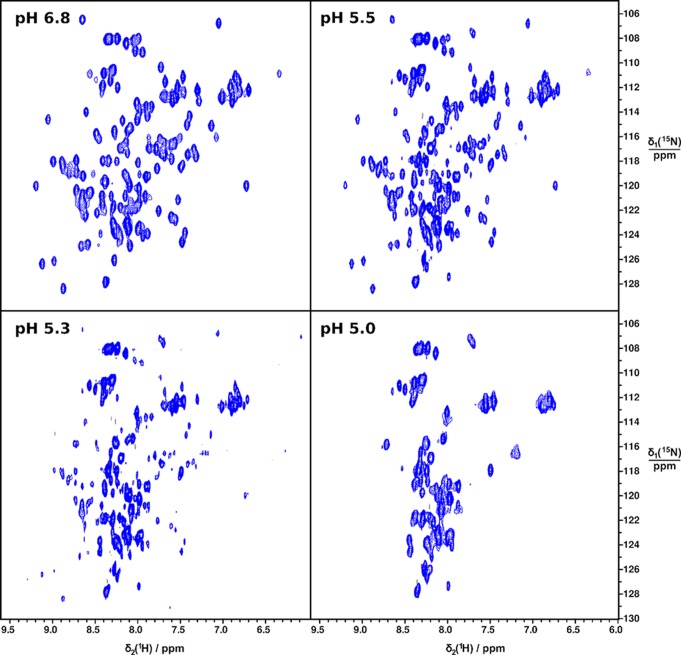
2D [^15^N-^1^H]-HSQC NMR spectra of CT at different pH.

**Figure 6:**
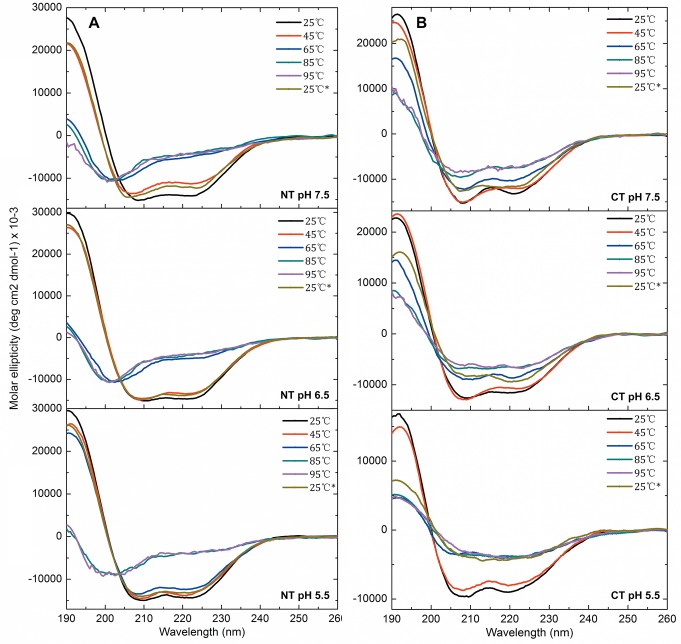
CD spectra of NT and CT. The residual molar ellipticity was measured at 25, 45, 65, 85, and 95°C and at 25°C after cooling for (A) NT and (B) CT at pH 7.5, 6.5, and 5.5 from top to bottom.

To address the third unresolved question—that is, how the terminal domains are affected by the conditions in the duct at a molecular level—we first compared the in vitro structural stability of NT and CT in the broad pH gradient now observed. We studied isolated domains, and it may be that these domains behave differently in their natural context of full-length spidroins. However, we have observed that NT followed by five repeats behaves as the isolated domain in terms of pH-dependent dimerization [11]. Urea and temperature denaturation studies at different pH values were performed for recombinant NT and CT (Figures 3 and 4). The stability of NT towards urea remained largely unchanged between pH 7.5 and 6.5, but was significantly increased between 6.0 and 5.0 (Figure 3). We here analyzed a minor ampullate spidroin (MiSp) NT, which has not been studied before, but a similar pH effect was recently shown for a major ampullate spidroin (MaSp) NT [11]. This indicates that the structural effects now observed are applicable to spidroins from major and minor ampullate glands, in concordance with the observation of CA in major and minor ampullate, aggregate, and tubuliform glands (Figure 2). A similar effect as seen for stability towards urea was seen for NT thermal stability; that is, it was increased at lower pH (Figure 4). Dimerization of NT is completed at pH 6 [11], and the subsequent stabilization of NT dimers between pH 6 and 5 (Figure 3) may result in the firm locking of spidroins into multimers in the distal part of the duct (cf., Figure 1). CT, in sharp contrast to NT, was gradually destabilized towards urea (Figure 3) and temperature (Figure 4) when pH was lowered from 7.5 to 5.0. Heteronuclear single quantum coherence (HSQC) nuclear magnetic resonance (NMR) spectra of CT showed a folded structure at pH 6.8, whereas a gradual conversion to an unfolded state was observed at a pH below 5.5, and at pH 5.0, it is completely unfolded (Figure 5). Moreover, we observed that CT irreversibly converted from α-helical to β-sheet structure upon thermal denaturation at pH 5.5, but not at pH 6.5 or 7.5 (Figure 6 and Table S1). The fact that NMR spectroscopy of CT shows an unfolded state at pH 5.0 (Figure 5) whereas circular dichroism (CD) spectroscopy and urea denaturation shows residual structure at pH 5.0 (Figure 3) may be explained by the different CT concentrations (0.3 mM versus 5 µM) and recording times (hours versus minutes) used. It should also be pointed out that unfolded species should have increased NMR intensities (and may thus be overestimated relative to folded species) due to favorable relaxation and dynamic properties and that helical structure (which is observed by CD) may be present in the species that are observed as random coil/unfolded by NMR. Denaturation of NT, in contrast, resulted in mainly unordered structure and was reversible at all three pH values (Figure 6 and Table S1). It may be worth noting that the structural conversion now observed for CT, but not for NT, resembles that seen for the spidroin dope [18], which may be relevant for the trigger mechanism as discussed below.

**Figure 7:**
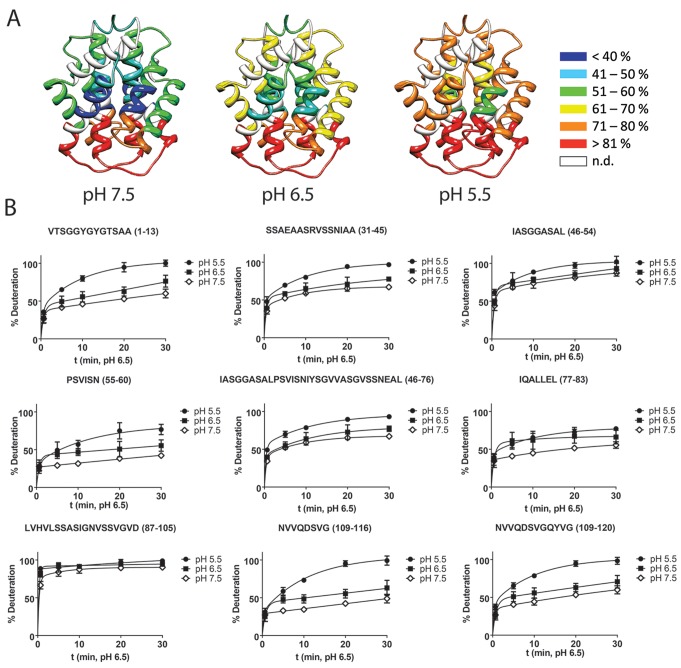
Summary of HDX ESI MS data for CT. (A) Deuterium incorporation in *A. ventricosus* MiSp CT at each pH. The degree of deuteration of the peptic peptides at pH 7.5, 6.5, and 5.5 is indicated according to the color code on the right. (B) Deuterium uptake graphs for the major peptic peptide species at pH 7.5, 6.5, and 5.5. Graphs show the average of three repeats. The error bars indicate the standard deviations. The corresponding peptic peptide sequences are given above each graph.

**Figure 8:**
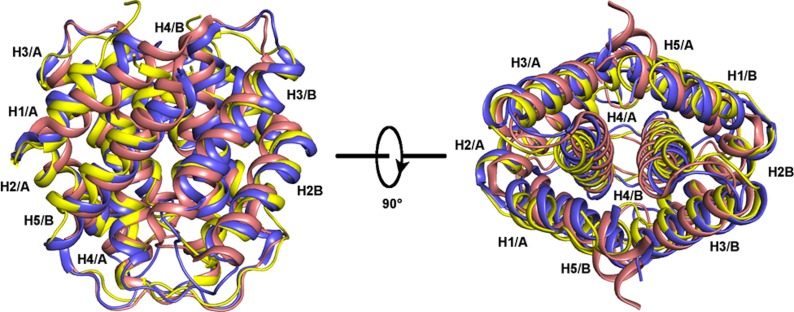
Superposition of MiSp CT structures from *A. ventricosus* (yellow) and *N. antipodiana* (blue, PDB code 2M0M) and the MaSp CT structure from *A. diadematus* (pink, PDB code 2KHM). Helices are shown as ribbons and labeled H1–H5. The letter A/B indicates the subunit.

Next, we used hydrogen-deuterium exchange mass spectrometry (HDX-MS) to study the backbone conformational dynamics of CT at pH 7.5 to 5.5. No major differences in HDX were seen between pH 7.5 and 6.5, but helices 2, 3, and 5 showed increased HDX at pH 5.5 compared to at pH 6.5 (Figure 7), indicating increased structural flexibility at lower pH. Previous studies of CT [4],[19] have identified a strictly conserved salt bridge between an Arg residue in helix 2 and a Glu residue in helix 4. The NMR structure of *Araneus ventricosus* MiSp CT now studied (Figure 8 and Table S2) is very similar to those of MiSp CT from *Nephila antipodiana* [19] and MaSp CT from *A. diadematus* [4] with backbone root-mean-square deviations (RMSDs) of 2.4 Å and 3.4 Å, respectively (over 202 residues from both chains; see Figure 8). Largest differences are observed for the N-terminal helix, which is shorter, and the C-terminal helix, which is kinked near the C-terminus in the *A. ventricosus* MiSp CT structure. A salt bridge between Arg38 in H2 and Glu82 in H4 is indeed found in *A. ventricosus* MiSp CT (Figure 8). Computational pKa predictions [20] of the available CT structures uniformly suggested that the Glu residue in H2 (that participates in the saltbridge) has a pKa ≥6, making it possible to protonate in the pH interval now observed in the gland, and mutations interfering with this salt bridge greatly destabilize CT [4],[19]. Our results suggest that protonation of the conserved Glu in H2 is involved in pH-dependent unfolding of CT in spider silk glands, and further experimental studies are warranted to determine exactly what residues are protonated in CT at low pH. Although the NMR structures of several CTs from different spidroins have been solved and their biochemical properties have been studied, the now observed pH responsive behavior of this domain has not been investigated in detail before [4],[5],[19],[21]–[23]. The shared overall fold suggests a conserved function of CT, but the possibility that CT has diverse functions in different silks cannot be excluded and is an important topic for further studies.

**Figure 9:**
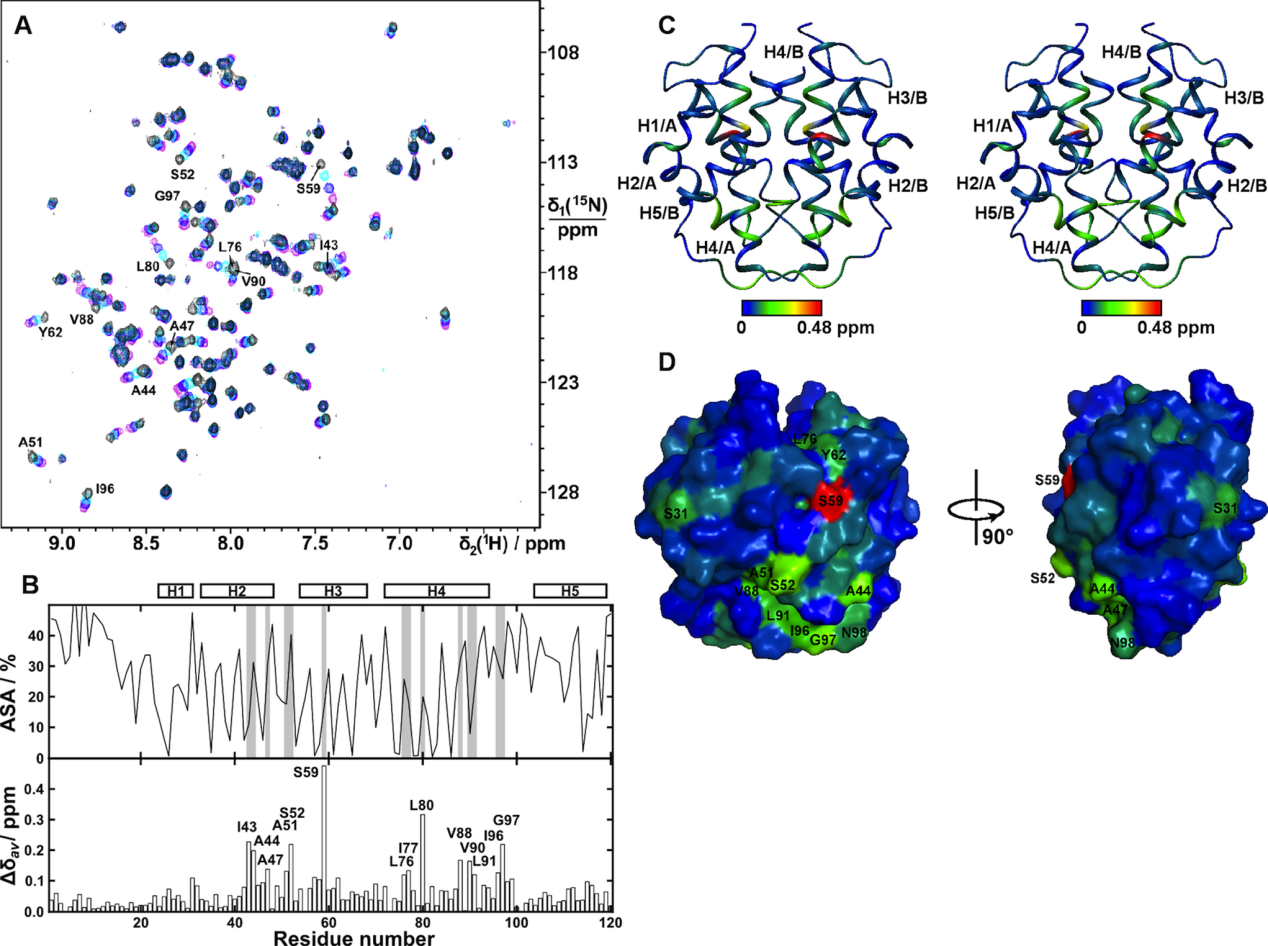
CT interacts with CS_2_. (A) Overlay of 2D [^15^N-^1^H]-HSQC NMR spectra of MiSp CT with CS_2_ added to concentrations of 0 mM (magenta), 50 mM (blue), 100 mM (cyan), and 200 mM (black). (B, Top) Accessible surface area (ASA) for individual amino acid residues, expressed as percentage of the total surface area of each residue. The most perturbed regions are shaded in grey. (B, Bottom) Chemical shift perturbations of MiSp CT backbone amides upon addition of CS_2_ (0 to 200 mM). The most perturbed residues are labeled and positions of helices 1–5 are indicated above the plot. (C) Stereoview of the MiSp CT structure, color-coded to reflect the backbone amide chemical shift perturbations of (B). (D) Surface view of *A. ventricosus* MiSp CT, color-coded to reflect the backbone amide chemical shift perturbations from (B). The surfaces of the most perturbed residues are labeled.

**Figure 10:**
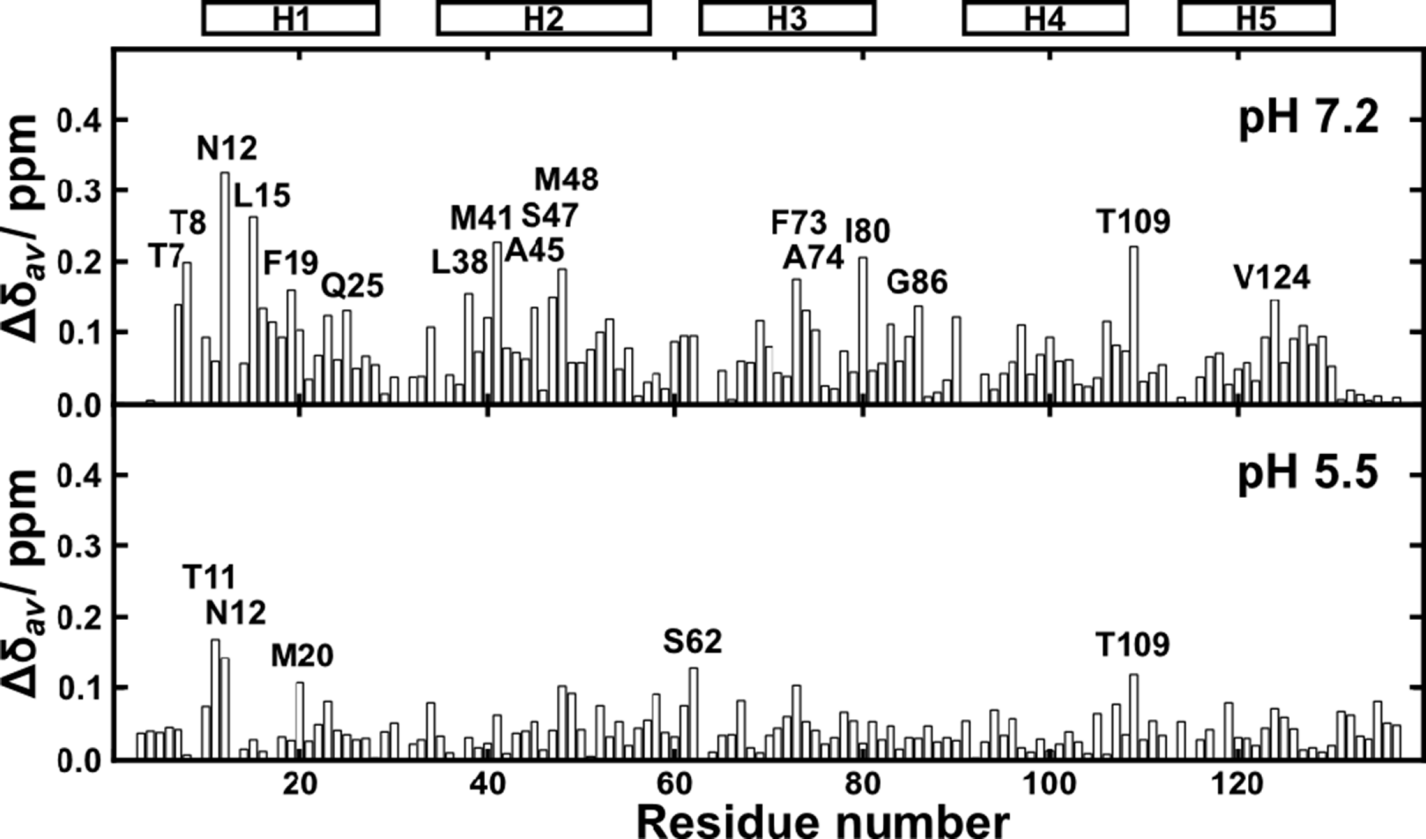
CS_2_ effects on NT. Chemical shift perturbations of MaSp NT backbone amides at pH 7.2 and 300 mM NaCl (upper panel) and pH 5.5 (lower panel) upon addition of CS_2_ (0 to 200 mM). The most perturbed residues are labeled, and positions of helices 1–5 are indicated above the plot.

The conditions now determined for the distal parts of the gland—that is, low pH combined with increasing HCO_3_^−^ concentration and low CO_2_ permeability of the gland—imply that pCO_2_ is elevated along the sac and duct. For MaSp CT, it has been shown that shear forces induce conformational changes that result in increased exposure of nonpolar surfaces [4], and CO_2_ interacts mainly with nonpolar regions in proteins [24],[25]. Therefore, we used the CO_2_ analogue CS_2_ [24] to identify potential interaction sites in the NMR structure of *A. ventricosus* MiSp CT. CS_2_ interacts specifically with a few, mainly hydrophobic, CT residues distributed in helices 2–4, of which many are partly buried (Figure 9A–D). NT on the other hand shows weak interactions with CS_2_ and only at conditions that favor the monomeric form, at pH 7.2 and 200 mM salt (Figure 10), which is characteristic to parts of the gland where pCO_2_ is low (Figure 1). In contrast to CT, no specific interactions between NT and CS_2_ were found at pH 5.5 (Figure 10), suggesting that NT stabilization at low pH (Figure 3 and Figure 4) protects its hydrophobic, buried residues from interacting with CO_2_.

**Figure 11:**
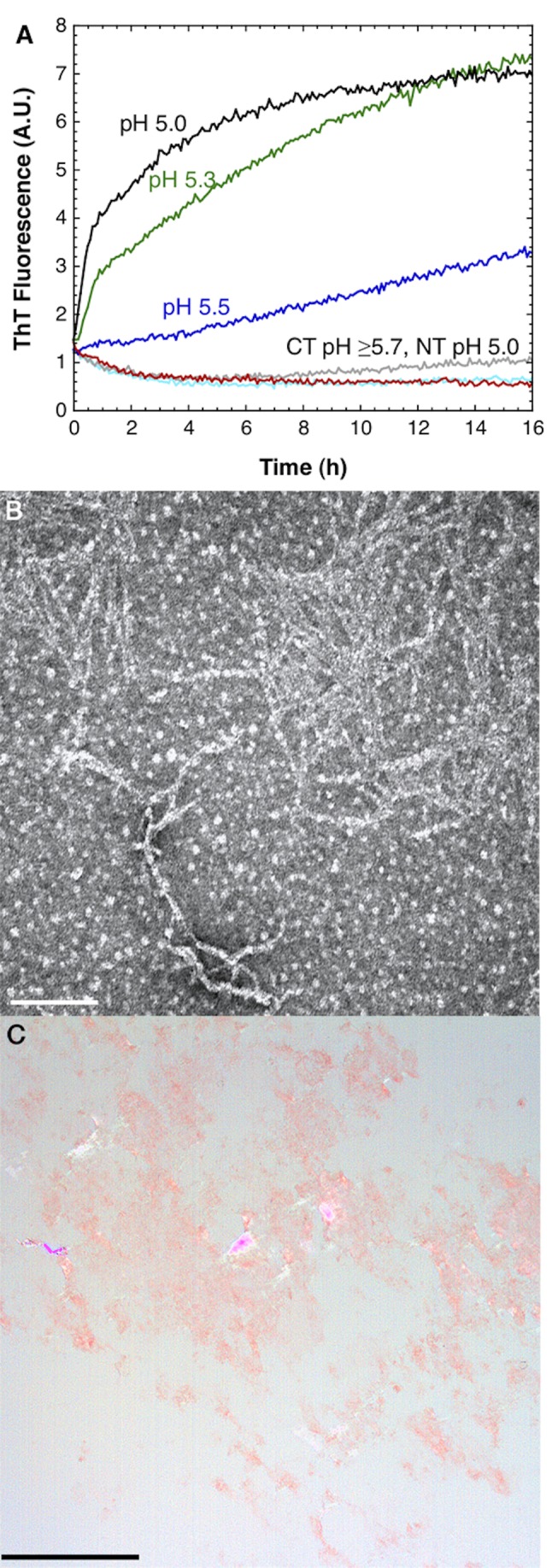
CT forms amyloid-like fibrils at low pH. (A) ThT fluorescence for CT at pH 6.0, 5.7, 5.5, 5.3, and 5.0, and NT at pH 5.0, in sodium acetate buffer. (B) Transmission electron micrograph showing fibrils formed from CT incubated at pH 5.5. Scale bar, 200 nm. (C) Congo red stained fibrils formed by CT at pH 5.5, viewed under crossed polarizers. Green birefringence is visible. Scale bar, 50 µm.

Amyloid fibrils are β-sheet polymers formed from (partly) unfolded proteins in a nucleation-dependent reaction and are found in tissue deposits associated with disease but also in some functional protein aggregates [26]. Amyloid fibrils share similarities with the β-sheets of spider silk and have been observed in the distal third of the spinning duct by electron microscopy (EM), and it was proposed that the spidroin repetitive parts are responsible for the amyloidogenic behavior [27]. The poly-Ala segments of spidroins need to rapidly form β-sheet structure in silk formation, although Ala is highly prone to form α-helices [28], raising the question, What nucleates this process? We investigated whether CT may convert to amyloid-like fibrils at low pH by measuring Thioflavin T (ThT) fluorescence of CT over time at different pH values. When ThT binds to β-sheet polymers in amyloid-like fibrils, it gives an increased fluorescence [29]. At pH 5.5 and below, CT converted to a ThT-positive state, which was not observed at higher pH, or for NT at any pH tested (Figure 11A). Analysis of the ThT-positive aggregates by transmission EM showed typical amyloid-like fibrils, 5–10 nm thick, elongated and nonbranched (Figure 11B). Only samples of CT incubated at pH 5.5 showed the presence of amyloid-like fibrils. Furthermore, the CT fibrils were positive for Congo red staining and showed green birefringence under polarized light (Figure 11C), another hallmark of an amyloid-like fiber [30].

The spidroins' terminal domains are highly conserved, both between species and between different types of silks [31], which suggest that they play important roles in spider silk formation rather than for the silks' mechanical properties. Further supporting the hypothesis of general polymerization mechanisms between different types of silks, CA is found in the distal parts of several different spider silk glands and occur at the same location as the observed structural changes of NT and CT will take place provided that their behavior *in vitro* is recapitulated *in vivo*. NT and CT are unique to spidroins and there are no known homologues. The lock (accomplished by NT) and trigger (accomplished by CT) mechanism proposed herein is therefore likely unique for spider silk formation, in contrast to the previously identified shear-induced polymerization mechanism that also apply to, for example, silk worm silk formation [32]. A detailed understanding of the natural spinning process will be vital for the development of a spinning process capable of generating truly biomimetic spider silk fibers and may provide novel insights into Nature's way of confining amyloid fibril formation to a specific location.

In summary, the spidroin N- and C-terminal domains show synchronous and opposite structural changes in response to the physiological conditions of the spinning duct. CT unfolds into β-sheet nuclei that can trigger rapid polymerization of the spidroins, whereas gradually locked NT dimers alleviate the need for rapid diffusion [11],[33], firmly interconnect the spidroins, and allow for propagation of pulling forces along the peptide chains. These events are driven by CO_2_ and proton gradients that ensure temporal and spatial confinement of the divergent structural changes of CT and NT. This novel lock and trigger mechanism elegantly explains how silk formation can occur at a very high speed, more than 1 m/s [34], and at the same time be confined to the very distal part of the spinning duct.

## Materials and Methods

### Preparation of Concentric ISMs to Measure pH, CO_3_^2−^, Na^+^, K^+^, and Cl^−^

Concentric ISMs [12] were used to measure the concentrations of hydrogen, carbonate, sodium, potassium, and chloride ions. Thin-walled borosilicate glass capillaries of two different diameters were used for construction of concentric ISMs. The capillary forming the outer barrel (outer diameter 2.0 mm, inner diameter 1.5 mm, A-M Systems 6185) was pulled to a tip diameter of 2–4 µm using a Flaming/Brown micropipette puller (Sutter Instrument Co. US, Model P87). The tip of the outer barrel was silanized by back-filling with *N,N*-dimethyltrimethylsilylamine (Fluka 41716), after which the barrel was mounted on a micromanipulator and heated using a hot air gun giving temperatures of 200–300°C for 60 s. Ion-selective cocktails for H^+^ (Fluka 95291), CO_3_^2−^ (described by Chesler et al.) [35], Na^+^, K^+^, and Cl^−^ were sucked into the tip to form a 100 to 200 µm long column, and a backfilling solution (pH electrode, 150 mM NaCl pH 7.4; CO_3_^2−^ electrode, 10 mM NaHCO_3_, 150 mM NaCl) was added in the middle of the outer barrel. The inner barrel (outer diameter of 1.2 mm and inner diameter of 0.9 mm, A-M Systems 6160) was pulled to a tip diameter of 1 µm and filled with 3 M KCl pH 7.4. The inner barrel was then inserted into and secured in the outer barrel, the inner glass tip being positioned 4–10 µm away from the outer barrel tip. A silver wire was inserted into the inner barrel and connected to an amplifier.

The ISMs were calibrated using pH 6.87 and pH 7.42 buffers, 50, 100, 200, and 400 mM Na^+^ or Cl^−^, or using 1, 2, 4, and 8 mM K^+^, respectively. Carbonate electrodes were calibrated as described [35].

### ISM Measurements of Major Ampullate Glands

Adult female *N. clavipes* collected in Florida from September to November were kept in individual containers and fed water. Spiders were anaesthetized with CO_2_ gas before severing at the pedicle. Dissection of the major ampullate glands was carried out in a modified spider Ringer [36] (with 2 mM MgCl_2_, 2 mM CaCl_2_, 3 mM KCl, and 10 mM glucose) buffered with 26 mM bicarbonate and 5% CO_2_, yielding a pH of 7.4.

Major ampullate glands were mounted in a submersion-style incubation chamber and superfused with HCO_3_^−^ and CO_2_-buffered modified spider Ringer at room temperature. ISM measurements were performed in triplicates in different parts of the gland. The difference in potential between the bath and the inside of the gland was recorded on a chart recorder (Zipp and Konnen) and later translated into change in concentration of the ion of interest using the Nernst equation (H^+^, Na^+^, K^+^, Cl^−^) or a modified Henderson–Hasselbalch equation [35] (CO_3_^2−^) to get the concentration of HCO_3_^−^. Determined pH values and HCO_3_^−^ concentrations were used to calculate pCO_2_ according to the Henderson–Hasselbalch equation, assuming equilibrium.

To study the influence of CA activity on the pH gradient, some glands were incubated for 1 h in 0.1 mM methazolamide (M4156, Fluka), a membrane-permeable CA inhibitor, prior to pH measurements, after which the methazolamide was washed away for 30 min and pH measurements repeated.

Some glands were subjected to CO_2_ permeability studies. Glands were dissected, mounted, and superfused with HCO_3_^−^ and CO_2_-buffered spider Ringer at room temperature as described above. A pH electrode was inserted into the gland, after which the surrounding Ringer solution was buffered by 26 mM bicarbonate and 100% CO_2_. pH measurements were continued up to 1 h to see if intraluminal pH changed in response to the elevated pCO_2_ surrounding the gland. The Ringer solution was then changed again, being buffered by 26 mM bicarbonate and 5% CO_2_, yielding a pH of 7.4, after which the pH electrode was removed from the gland and put in the Ringer and pH was recorded. This was made to ensure that the electrode had not been drifting.

### Histochemical Localization of CA Activity

Spiders (*A. diadematus*, *N. clavipes*, *E. australis*, and *Tegenaria sp.*) were anesthetized and sacrificed as described above. Dissection was carried out in 67 mM sodium phosphate buffer at pH 7.2 or in a modified Spider Ringer (see above). Some opisthosomas were fixed and embedded directly after removal of the exoskeleton, whereas others were dissected so that the major and minor ampullate glands could be isolated before fixation.

Tissues for histochemical localization of CA activity were immersion fixed in 2.5% (v/v) glutaraldehyde in 67 mM phosphate buffer, pH 7.2, for 24 h at 4°C and subsequently rinsed with phosphate buffer, pH 7.2. After fixation, tissues were dehydrated using increasing concentrations of ethanol, infiltrated and embedded in a water-soluble glycol methacrylate (Leica Historesin embedding kit).

Historesin embedded major and minor ampullate glands and opisthosomas were sectioned at 2 µm in a microtome (Leica RM 2165) and stained for CA activity using a histochemical method [14]. The method involves incubation of sections in a medium containing NaHCO_3_, CoSO_4_, H_2_SO_4_, and KH_2_PO_4_, whereby carbon dioxide leaves, pH increases, and a cobalt–phosphate–carbonate complex is formed at sites with CA activity. This complex is then converted into a black cobalt–sulphide precipitate. The sections were counterstained with Azure blue. For control of unspecific staining, the CA inhibitor acetazolamide was included in the incubation medium.

### Protein Expression and Purification

*A. ventricosus* MiSp NT and CT coding gene fragments corresponding to (NT: GSGNSQPIWT NPNAAMTMTN NLVQCASRSG VLTADQMDDM GMMADSVNSQ MQKMGPNPPQ HRLRAMNTAM AAEVAEVVAT SPPQSYSAVL NTIGACLRES MMQATGSVDN AFTNEVMQLV KMLSADSANE VST) and (CT: GSGNSTVAAY GGAGGVATSS SSATASGSRI **VTSGGYGYGT SAAAGAGVAA GSYAGAVNRL SSAEAASRVS SNIAAIASGG ASALPSVISN IYSGVVASGV SSNEALIQAL LELLSALVHV LSSASIGNVS SVGVDSTLNV VQDSVGQYVG**) were amplified by PCR with the full-length MiSp gene as template [37], cloned into a modified pET vector (resulting in the target proteins being fused to His tag–Thioredoxin–His tag followed by a thrombin cleavage site) and transformed into BL21 (DE3) *Escherichia coli*. The *E. coli* were grown at 37°C in LB medium containing 70 mg/l kanamycin until OD_600_ was about 0.9. The temperature was lowered to 30°C, IPTG was added to a final concentration of 0.3 mM, and the cells were incubated for about 4 h. The *E. coli* were then harvested by centrifugation at 6,400×*g* for 20 min at 4°C (Sorvall RC 3BP+, 500 ml flasks), after which the pellet was resuspended in 20 mM Tris pH 8.0, 1 mg/ml lysozyme was added, and the solution was incubated on ice for 30 min. Next, DNase and MgCl_2_ were added and the mixture was kept on ice for 30 min. The cell lysate was centrifuged (27,000×*g*) at 4°C for 20 min (centrifuged as above, 50 ml tubes). For purification of CT, the supernatant was loaded on a Ni-NTA column and the fusion protein was eluted with 300 mM imidazole. For purification of NT, which is mainly found in the pellet after lysis, pellets were resuspended in 20 mM Tris pH 8.0 containing 2 M urea, sonicated for 2 min, and the supernatant was treated as for CT. The fusion proteins were then dialyzed against 20 mM Tris pH 8.0 overnight at 4°C, cleaved by 1/1,000 (w/w) thrombin, and run over a Ni-NTA column to remove the fusion tag. This resulted in essentially pure NT or CT (>90% purity as determined by SDS PAGE gel electrophoresis and Coomassie staining).

For NMR structure determination, we initially expressed a 150-amino-acid-residue-long C-terminal part of *A. ventricosus* MiSp (full-length sequence above). The expressed protein was labeled with ^15^N, and the NMR spectrum showed that the first 25 residues adopt random coil fold. Therefore, *A. ventricosus* MiSp CT was truncated and residues 31–150 (marked in bold in the sequence above) were expressed in minimal medium and labeled by ^15^N and ^13^C/^15^N. The NMR sample was prepared by adding 8% (v/v) D_2_O and 0.02% (w/v) NaN_3_ to a 1 mM solution of uniformly ^13^C/^15^N-labelled protein in 20 mM sodium phosphate buffer (pH 6.8) with 20 mM NaCl.

### NMR Measurements

All NMR experiments were carried out at 298 K on a 600-MHz Varian Unity Inova spectrometer equipped with an HCN triple-resonance pulsed-field-gradient cold probe. The following 2D and 3D spectra were acquired for backbone resonance assignment (number of complex points given in parentheses): [^15^N-^1^H]-HSQC (1024×128), HNCA (1024×24×40), CBCA(CO)NH (2048×48×40), HNCO (1024×24×40), HN(CA)CO (1024×24×40), and for side-chain assignment and NOE restraint collection (mixing time given in parentheses): ^15^N-resolved NOESY-HSQC (1024×38×150, 60 ms), ^13^C(aliphatic)-resolved NOESY-HSQC (768×52×150, 60 ms), and ^13^C(aromatic)-resolved NOESY-HSQC (1024×16×150, 60 ms). Additionally, in order to identify intermolecular NOEs, a ^13^C/^15^N-filtered ^13^C(aliphatic)-resolved NOESY-HSQC spectrum (768×34×70, 60 ms) was recorded on a sample containing 50% ^13^C/^15^N-labelled and 50% unlabelled proteins [38] that was prepared by mixing equal amounts of labeled and unlabelled proteins in 8 M urea followed by dialysis against the NMR sample buffer. The same sample was afterwards used to probe interactions with CS_2_. Aliquots of 20% CS_2_ in DMSO were added in a stepwise manner to the NMR sample of CT, yielding CS_2_ concentrations of 50 mM, 100 mM, and 200 mM, and a 2D [^15^N-^1^H]-HSQC spectrum was recorded each time. To account for perturbations due to DMSO, a reference experiment was performed by adding DMSO only in the same amounts. CS_2_-induced chemical shift perturbations were calculated by comparing the spectrum at 200 mM CS_2_ with the spectrum at the end of the reference titration with DMSO, and using the formula (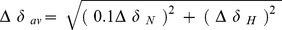) [39]. All spectra were processed with Bruker TopSpin 3.1 and analyzed using CARA [40]. The assigned chemical shifts have been deposited in BioMagResBank (accession number 19579). To probe interactions between NT and CS_2_, NT from MaSp1 from *E. australis* was expressed and purified as previously described [10]. Chemical shift perturbations of MaSp NT backbone amides were determined at pH 7.2 and 200 mM NaCl and at pH 5.5 upon addition of CS_2_ (0 to 200 mM) as described for CT.

For 2D [^15^N-^1^H]-HSQC NMR spectra of MiSp CT, samples at pH 5.0, 5.3, and 5.5 were prepared by diluting 50 µl of a concentrated stock solution of MiSp CT in 20 mM sodium phosphate buffer, 20 mM NaCl, 0.03% NaN_3_, pH 6.8 with 200 µl of 100 mM CD_3_COOD/CD_3_COONa, 20 mM NaCl, 0.03% NaN_3_ buffer, and adding 20 µl of D_2_O.

### Structure Calculation

Automated peak picking of the three NOESY spectra was performed using UNIO-ATNOS/CANDID 2.0.2 [41]. Distance restraints were obtained from these peak lists using the internal NOE calibration procedure of CYANA 2.1 [42]. Intermolecular contacts were identified by analysis of the ^13^C,^15^N-filtered NOESY spectrum and used as distance restraints with an upper limit of 5 Å. No explicit torsion-angle restraints were used in the input. Structure calculations were performed using CYANA 2.1 [42] and involved seven iterations of automated NOE assignment with the routine CANDID [41] followed by a simulated annealing procedure starting in the first cycle from a homology model generated based on the MiSp CT structure from *N. antipodiana* [19] (PDB accession code 2M0M) that was annealed in 15,000 steps of torsion-angle dynamics. This approach was used to reduce the assignment ambiguity during the first cycles of the automated NOE assignment and resulted in significantly more unambiguous distance restraints in the final cycle of the calculation concomitantly with a lower target function value. The 20 conformers with the lowest residual restraint violations were energy minimized in a water shell using the program CNS 1.2 [43],[44], and their coordinates were deposited in PDB (accession code 2MFZ). Table S2 shows an overview of the restraints used and structural statistics. Ramachandran statistics for structured part (residues 20–120) are 94.2% most favored, 5.8% additionally allowed regions; for all residues including the unstructured N-terminal tail, 88.3% most favored, 11.1% additionally allowed, 0.3% generously allowed, and 0.3% disallowed regions.

### ThT Fluorescence Measurements

For analysis of amyloid fibril formation, 10 µM of *A. ventricosus* MiSp NT and CT were incubated under quiescent conditions at 25°C with 10 µM ThT in 20 mM sodium phosphate or 50 mM sodium acetate buffer with or without 154 mM NaCl at different pH values between 5.0 and 7.5. ThT fluorescence was recorded on a BMG FLUOstar Galaxy plate reader using bottom optics in 96-well polyethylene glycol-coated black polystyrene plates with a clear bottom (Corning Glass, 3881) using a 440-nm excitation filter and a 490-nm emission filter.

### Transmission Electron Microscopy

For analysis of amyloid fibrils, 10 µM of *A. ventricosus* MiSp NT and CT were incubated overnight (12–16 h) under quiescent conditions at 25°C in 20 mM sodium phosphate buffers with or without 154 mM NaCl at pH 7.5, 6.5, and 5.5, respectively. Samples were incubated overnight and 2 µl were adsorbed on copper grids, stained with 2.5% uranyl acetate in 50% ethanol for about 20 s, and examined and photographed with a Hitachi H7100 electron microscope at 75 kV.

### Congo Red Staining

Ten µM *A. ventricosus* MiSp CT was incubated at 37°C with shaking (250 rpm) for 2.5 h at pH 5–7 in 20 mM sodium phosphate and 50 mM sodium acetate buffers, respectively. Samples were centrifuged, supernatant removed, washed with dH_2_O, and then centrifuged again. The supernatant was removed and 10 µl dH_2_O was added, the sample was vortexed, and droplets (0.8 µl) were applied to microscopical slides, air dried, and stained with Congo red B [45]. After mounting under coverslips, the materials were examined in a polarization microscope for Congophilia and green birefringence.

### Urea Denaturation

*A. ventricosus* MiSp NT and CT stability between pH 5.0 and 7.5 with and without 154 mM NaCl was determined by urea denaturation. Like in previous denaturation studies of MiSp CT from *N. antipodiana* [19], we used a two-state model for analyzing our denaturation data. Although a two-state transition is supported by a CD isodichroic point at 203 nm [46] for NT at low pH (Figure 4A), this is not the case for CT at any pH, or NT at pH 7.5 (Figure 4B). To emphasize that we assumed a two-state transition for both NT and CT, the urea concentrations derived from fitting the data to a two-state unfolding model are referred to as apparent half-denaturation ([den]^50%^). Notably, the main conclusion from these experiments—that NT and CT respond in completely opposite ways to lowered pH—is not dependent on whether a two-state transition applies or not.

For NT, urea-induced denaturation was performed by diluting the protein to 5 µM in 20 mM HEPES/20 mM MES with 0–7 M urea in 0.25 M steps. Tryptophan fluorescence emission spectra were measured on a spectrofluorometer (Tecan Safire 2) using Costar black polystyrene assay plates with 96 flat bottom wells. The samples were excited at 280 nm using a 5 nm bandwidth, and emission spectra were recorded in 1 nm steps between 300 and 400 nm using a 10 nm bandwidth. Spectra were recorded at constant pH values ranging from 5.0 to 7.5 with 0.2–0.5 unit steps. For CT, CD spectroscopy at 222 nm was used to determine [den]^50%^ as a function of pH. The CT samples were diluted to 7.5 µM in 20 mM sodium phosphate buffer and ran with 0–7 M urea in 0.25 M steps. At each pH, the average 222 nm CD ellipticity from three temperature scans for different urea concentrations were obtained with the settings described below (see CD spectroscopy). The ellipticities for each measured pH values ranging from 5.0 to 7.5 with 0.2–0.5 unit steps were plotted against the urea concentration and fitted to a two-state unfolding model in order to determine the [den]^50%^ by KaleidaGraph.

### CD Spectroscopy

CD spectra were recorded from 260 to 190 nm at 25°C in 0.1 mm path length quartz cuvettes using an Aviv 410 Spectrometer. The wavelength step was 0.5 nm, averaging time 0.3 s, scan speed 20 nm/min, time constant 100 ms, and bandwidth 1 nm. The spectra shown are subtracted for background and averaged over three consecutive scans. The HT voltages were always below 600 V during the entire scans. Spectra of 7.5 µM *A. ventricosus* MiSp NT (110 µg/ml) or CT (90 µg/ml) in 20 mM sodium phosphate buffer at pH 7.5, pH 6.5, or pH 5.5 were recorded at 25, 45, 65, 85, and 95°C and at 25°C again after cooling. For temperature melting curves, the CD at 222 nm was monitored between 25 and 95°C with 1°C/min increase.

### Electrospray Ionization Mass Spectrometry (ESI MS) and HDX

Deuteration buffers were prepared by freeze-drying 200 µl of 20 mM sodium phosphate buffer, pH 5.5 or 6.5, followed by reconstitution in 200 µl D_2_O (Cambridge Isotopes). *A. ventricosus* MiSp CT was diluted from 555 µM stock solution, pH 6.5, to 55.5 µM in deuterated phosphate buffer, pH 6.5 or 5.5. We removed 19.5 µl aliquots after 400 s, 50 min, 100 min, 200 min, or 300 min (pH 5.5) or after 40 s, 5 min, 10 min, 20 min, or 30 min (pH 6.5). Aliquots were placed in prechilled 500 µl Eppendorf tubes containing 0.5 µl 5% TFA (Merck), vortexed, and immediately frozen in liquid nitrogen. For a fully deuterated control, CT was incubated in deuterated phosphate buffer, pH 6.5, for 24 h at 25°C. Samples were stored at −80°C until ESI MS analysis.

Samples were thawed and immediately injected into an HPLC system using a chilled 25 µl Hamilton syringe. CT protein was digested in a Porozyme pepsin cartridge (Applied Biosystems), and peptides were trapped and desalted in a Waters Symmetry C_18_ trap column (Waters). Two 140D solvent delivery systems (Applied Biosystems) were employed, operating at 20 µl/min (for washing with 0.05% TFA) or at 15 µl/min (for elution with 70% acetonitrile, 0.2% formic acid). Digestion and desalting were carried out in a single step for 10 min, and peptides were then eluted in a single step and delivered to the mass spectrometer via a TaperTip emitter (Proxeon). The entire flow system was submerged in an ice bath.

ESI spectra were acquired in positive-ion mode with a Waters Ultima API mass spectrometer (Waters) equipped with a Z-spray source. The source temperature was 80°C, the capillary voltage was 2.5 kV, and the cone and radiofrequency lens 1 potentials were 100 and 38 V, respectively. The mass spectrometer was operated in single-reflector mode to achieve a resolution of 10,000 (full width at half maximum). The mass scale was calibrated using [Glu1]fibrinopeptide B. Peptic peptides were identified based on a map of pepsin-digested undeuterated CT using automated liquid chromatography–tandem mass spectrometry (LC-MS/MS) analysis with a Waters NanoAcquity system (Waters). Peptide sequences were identified by individual analysis of collision-induced dissociation (CID) spectra using the Waters MassLynx and ProteinLynx software packages (Waters).

### Size Exclusion Chromatography

We analyzed 100 µl of 1 mg/ml *A. ventricosus* MiSp CT equilibrated 10 min in 20 mM HEPES/MES pH 7.5 or 5.5 using Sephacryl S-100 (GE Healthcare) in the same buffers and at a flow rate of 0.5 ml/min. Molecular mass standards aprotenin (6.5 kDa), ribonuclease (13.7 kDa), CA (29 kDa), ovalbumin (43 kDa), and conalbumin (75 kDa) were used for calibration.

## Supporting Information

Table S1 Percentage of different secondary structures estimated from the CD spectra of NT and CT at 25 and 95°C (Figure 6), using the Dichroweb server (http://dichroweb.cryst.bbk.ac.uk/html/home.shtml).
(PDF)

Table S2 NMR and refinement statistics for protein structures.
(PDF)

## Abbreviations

CA: carbonic anhydrase
CD: circular dichroism
CT: C-terminal domain
DMSO: dimethyl sulfoxide
EM: electron microscopy
ESI: electrospray ionization
HDX-MS: hydrogen-deuterium exchange mass spectrometry
HEPES: hydroxyethyl piperazineethanesulfonic acid
HSQC: heteronuclear single quantum coherence
ISM: ion selective microelectrode
LC-MS/MS: liquid chromatography–tandem mass spectrometry
Ma: major ampullate
MES: morpholineethanesulfonic acid
Mi: minor ampullate
MS: mass spectrometry
NMR: nuclear magnetic resonance
NT: N-terminal domain
pCO_2_: carbon dioxide pressure
Sp: spidroin
Spidroin: spider silk protein
ThT: Thioflavin T
