# Supplementary material for: Carbonic Anhydrase Generates CO2 and H+ That Drive Spider Silk Formation Via Opposite Effects on the Terminal Domains
Source: PLoS Biol. 2014 Aug 5;12(8):e1001921. doi: 10.1371/journal.pbio.1001921 (PMC4122339; doi:10.1371/journal.pbio.1001921)
Supplement: Table S1 — Percentage of different secondary structures estimated from the CD spectra of NT and CT at 25 and 95°C (Figure 6), using the Dichroweb server (http://dichroweb.cryst.bbk.ac.uk/html/home.shtml). (PDF) [file pbio.1001921.s001.pdf]

Table S1. Percentage of different secondary structures estimated from the CD spectra of NT and CT at 25 and 95 °C (Fig. 6), using the Dichroweb server (http://dichroweb.cryst.bbk.ac.uk/html/home.shtml).

	Secondary structure	25°C	95°C
**pH 7.5 NT**	Helix	41.3%	9.2%
	Strand	10.9%	14.9%
	Turns	17.4%	10.6%
	Unordered	30.8%	64.9%
**pH 6.5 NT**	α-helix	41.9%	9.2%
	β-sheet	12.1%	16.9%
	Turns	17.6%	1.1%
	Unordered	29.3%	69.7%
**pH 5.5 NT**	α-helix	42.7%	3.1%
	β-sheet	12.3%	19.2%
	Turns	17.3%	4.1%
	Unordered	27.9%	75.8%
**pH 7.5 CT**	α-helix	41.3%	18.3%
	β-sheet	14.6%	22.0%
	Turns	18.3%	16.9%
	Unordered	27.7%	39.2%
**pH 6.5 CT**	α-helix	36.5%	12.7%
	β-sheet	16.4%	34.4%
	Turns	19.1%	19.5%
	Unordered	29.0%	23.5%
**pH 5.5 CT**	α-helix	29.0%	13.0%
	β-sheet	19.2%	40.2%
	Turns	22.2%	17.7%
	Unordered	29.3%	27.2%
