# Supplementary material for: Carbonic Anhydrase Generates CO2 and H+ That Drive Spider Silk Formation Via Opposite Effects on the Terminal Domains
Source: PLoS Biol. 2014 Aug 5;12(8):e1001921. doi: 10.1371/journal.pbio.1001921 (PMC4122339; doi:10.1371/journal.pbio.1001921)
Supplement: Table S2 — NMR and refinement statistics for protein structures. (PDF) [file pbio.1001921.s002.pdf]

Table S2. NMR and refinement statistics for protein structures

	***A. ventricosus* MiSp CT**
**NMR distance and dihedral constraints**	
Distance constraints	
Total NOE	6060
Intra-residue	1024
Inter-residue	5036
Sequential (|i-j| = 1)	1548
Medium-range (|i-j| < 4)	1815
Long-range (|i-j > 5)	971
Intermolecular	702
**Structure statistics**	
Violations (mean and s.d.)	
Distance constraints (Å)	0.0109 ± 0.0004
Max. distance constraint violation (Å)	0.38 ± 0.05
Deviations from idealized geometry	
Bond lengths (Å)	0.0035 ± 0.0001
Bond angles (°)	0.440 ± 0.007
Impropers (°)	1.241 ± 0.043
Average pairwise r.m.s.d.** (Å)	
Heavy	0.46 ± 0.07
Backbone	0.78 ± 0.07

**Pairwise r.m.s.d. was calculated for residues 20-120 of both chains among 20 refined structures.
